# The Impact of Heterogeneity and Awareness in Modeling Epidemic Spreading on Multiplex Networks

**DOI:** 10.1038/srep37105

**Published:** 2016-11-16

**Authors:** Marialisa Scatà, Alessandro Di Stefano, Pietro Liò, Aurelio La Corte

**Affiliations:** 1 University of Catania, Dipartimento di Ingegneria Elettrica, Elettronica e Informatica, Catania, 95125, Italy; 2 University of Cambridge, Computer Laboratory, Cambridge (UK), CB3OFD, UK

## Abstract

In the real world, dynamic processes involving human beings are not disjoint. To capture the real complexity of such dynamics, we propose a novel model of the coevolution of epidemic and awareness spreading processes on a multiplex network, also introducing a preventive isolation strategy. Our aim is to evaluate and quantify the joint impact of heterogeneity and awareness, under different socioeconomic conditions. Considering, as case study, an emerging public health threat, Zika virus, we introduce a data-driven analysis by exploiting multiple sources and different types of data, ranging from Big Five personality traits to Google Trends, related to different world countries where there is an ongoing epidemic outbreak. Our findings demonstrate how the proposed model allows delaying the epidemic outbreak and increasing the resilience of nodes, especially under critical economic conditions. Simulation results, using data-driven approach on Zika virus, which has a growing scientific research interest, are coherent with the proposed analytic model.

In hundreds years of history, a huge literature have been proposed to study epidemic spreading and dynamics^1^,^2^,^3^,^4^,^5^,^6^,^7^, involving several research fields and assuming a key role in the field of network science^8^,^9^,^10^,^11^,^12^,^13^,^14^,^15^. Among all the possible dynamic scenarios explored^16^, multiplex networks provide the best suited underlying network structure for the study of dynamical processes taking place within the same set of nodes, such as the spreading of infectious diseases on multiplex networks^17^,^18^,^19^,^20^,^21^. Furthermore, recently, the interplay between disease and awareness dynamics^22^,^23^,^24^,^25^ has gained a lot of interest, studying how individuals, aware of the potential spread of a certain disease, are able to take preventive measures protecting themselves. In most of these studies, it is explored the interesting interplay between awareness and epidemics when both phenomena compete using different layers of propagation^23^,^26^,^27^,^28^,^29^,^30^. Although a lot of works have exploited the framework of multiplex networks and studied the dynamics of the two spreading processes, awareness and disease, none of them has explored the realistic coevolution of the two processes in all the layers of a multiplex network. Some authors have underlined the key role of network heterogeneity in comparison with homogeneous cases^31^. Investigating the real-world scenario of an emerging disease raises the challenge of quantifying the impact of awareness on the complex dynamics of the epidemic outbreak. Human reactions and their resilience against a virus is the outcome of the individual interplay of multiple tiles of a mosaic, which embodies personality traits, relationships, knowledge and well-being. To capture also the high complexity of social interactions, we explore the dynamics on a multiplex network, adding an extra dimension of analysis and a more natural description for such systems^32^,^33^,^34^. Moreover, even more realistically, the coevolution of epidemic and awareness spreading on a multiplex network is linked with how different socioeconomic conditions, together with the awareness of the single nodes, affect the human susceptibility^35^. Awareness and the economic healthy of nodes can drive the system dynamics, altering the individual approach towards infection. The introduction of a preventive isolation strategy is a way to keep under control properly chosen nodes belonging to specific social categories, which are more sensitive and susceptible with regards to the infection and could be less aware and economically disadvantaged other than being, at the same time, also central in their communities. The isolation strategy can be interpreted as a temporary “social herd immunity”, lowering the infection rate of the isolated nodes and the population as well, driving them towards a deep awareness. This can help the network and health management authorities to improve consciousness about disease, providing a better understanding of what values can be realized by this kind of investigation^36^. The key difference between our isolation strategy and that one proposed in ref. 18 is that we isolate nodes in a preventive way, not as a result of infection. For all these reasons, as discussed in ref. 36, in our opinion one of the actual challenges of the network theory is targeted at focusing on the realization of human rights and the detection of more sensitive categories in epidemic terms, protecting both the nodes and the community as a whole. Our work aims to propose a novel model of epidemic spreading, introducing an heterogeneous susceptibility of the single nodes of the network and the concept of preventive isolation of nodes. To this aim, we consider the epidemic spreading model coevolving with the awareness spreading in all the layers of the same multiplex network, analyzing the joint effect on the resulting complex dynamics, where each node participates to both the processes simultaneously. To the best of our knowledge, for the first time we face the even more intriguing and novel issue which consists of studying the spreading of the two processes without separating and constraining each of the spreading processes to only one of the layers. In other words, we decide not to disjoint the two processes in single layers, but rather to explore the complex dynamics of their coevolution in order to define a scenario as realistic as possible and more coherent with the real nature of multiplex networks^33^,^37^,^38^. The study of the two dynamics on a multiplex network allows us highlighting the psychological dynamics of awareness, which is marked by cognitive limitation^39^,^40^ and then a re-ranking of news based on the emerging matters. Keeping a watchful eye on the news is in contrast with the natural memory leaks or fading of attention by human brain towards invisible enemies, such as viruses. For this reason, in our model, we consider the fading due to this decay of attention. We explore how phase transitions and epidemic threshold changes according to network structure and heterogeneous infection rate, which in turn depends on the nodes’ awareness in the multiplex network. This is a sort of interdependence between epidemic and awareness dynamics under different socioeconomic conditions. Furthermore, we adopt a data-driven approach, comparing the resulting dynamics in a realistic context, which includes heterogeneous real data, taken from different sources. In particular, we deal with statistic estimators and markers of social and economic critical issues. These estimators allow us evaluating how the various characteristics of the node, also in terms of social categories, can affect the awareness and hence the epidemic dynamics. We compare analytic and simulation results using data observed in a particular temporal window^30^, to evaluate the coherence of our model fitted with data. The data are referred to Zika virus^41^, an emerging viral disease representing a present public health threat. It can be seen in several countries and it is currently raised as an international concern which is attracting the interest of interdisciplinary research^42^. As authors underline^43^, a major concern associated with Zika virus is the observed incidence of microcephaly in fetuses born to mothers infected with Zika. Given this evidence regarding Zika virus in pregnant women, this means that there is a strong and biologically coded attention by women on the fetuses and then the infants conditions. Such attention is high in men as well following the biological well-known questions of kin-directed altruism and reciprocal altruism. In our model, this is reflected in the fading rate, which will be naturally low in case of Zika virus infection since, in this case, awareness has a strong memory effect on pregnant women reducing their cognitive limitation^39^,^40^ as they have a large attention period (at least equal to pregnancy period). The analysis in terms of awareness is facilitated by the available evidence regarding the spectrum of Zika virus, producing a strong and widespread impact on dynamics^44^. The interest towards Zika is also due to the presence of two infection dynamics, from mosquitoes to humans and by sexual transmission. Our interest is to better understand to which extent the study of the coevolution of epidemic and awareness spreading on a multiplex network can change, even if only slightly, the spreading dynamic, delaying the virus outbreak and providing a temporal window where specific and strategic interventions can be scheduled.

## Results

### Discussing the applicability of SIR on realistic epidemiological models

Our model is a SIR-like model, thought as a “composed” SIR, that is an extension of the classic “Susceptible-Infected-Recovered” (SIR) epidemic model^1^,^10^,^45^, where we provide the hypothesis of heterogeneous susceptibility and a strategy of preventive isolation, which consists of preventively isolating a set of properly chosen nodes, based on the coevolution of the two processes on network, and structural properties of the multiplex network. To apply this model to Zika or other mosquito-borne pathogens^46^,^47^,^48^, rather than using the Ross-Macdonald models^49^, we need to face and reconcile the limiting assumptions of the SIR, e.g. the distinct lag between human and mosquito infection. Despite this discrepancy between assumptions of the SIR model and the reality of many pathogen systems, as observed in ref. 41, our model fits mathematically with the nature of pathogen in epidemic terms. The applicability of the proposed SIR-like model is related with the Zika virus transmission since it can be transmitted in different ways^50^. Among them, since one of our targets is to explore the large scale transmission of Zika virus, the human-to-mosquito-to-human cycle transmission is the unique type of transmission we are interested in, which justifies the choice of a SIR-like as an abstract diffusion model^41^. We chose to consider the compartment “Recovered” as a state linked to comorbidities and in particular to Guillain-Barrè syndrome (GBS)^51^. Among the various comorbidites with Zika virus (e.g. microcephaly), the GBS is the most relevant in considering the choice of SIR-like model with a “recovered” state. The phenotipic effects range from a few days to six months, but on average these effect last about one week. Therefore, under the hypothesis of a six-months recovery period, individuals can be assumed phenotipically healthy in this time span. There is a non-zero probability to be reinfected but, being GBS very rare, we can reasonably assume that there is not a significative likelihood of reinfection in the specified time span. Considering the socioeconomic clustering population based, in each cluster we can apply a different *SIR* model. Events and social phenomena, such as Rio Olympics or Carnival in Brazil, are synchronization factors which break the symmetries and shuffle both travelers and native populations, transforming and randomizing SIR models of each cluster into a globally synchronized SIR^52^. Moreover, these events may produce a resonance effect causing another peak of infection shifted in time and space, due to subsequent sexual contacts and spread of behaviors among social contacts^53^. As assessed in ref. 54, “..we are not going to know the full impact of this epidemic for several more months until we see whether additional waves of microcephaly cases are born”.

### Model

**Figure 1:**
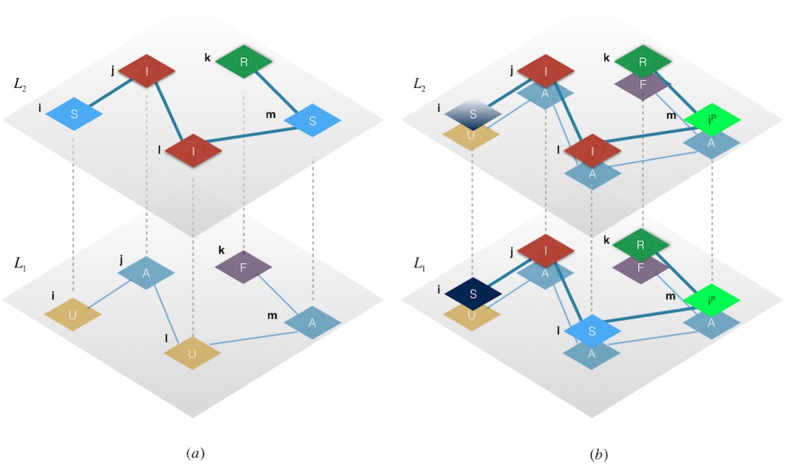
Schematic example of the coevolution of epidemic and awareness spreading on a multiplex Network. The multiplex network is made of N = 5 nodes embedded within M = 2 layers, each one containing 4 links. Nodes are indicated by a letter corresponding to their states (*S* = susceptible, *I* = Infected, *R* = Recovered, *U* = Unaware, *A* = Aware, *F* = Faded, *i*^*p*^ = Preventive isolated) in the epidemic and awareness spreading process. The dashed lines represent inter-layer connections, while the continuous lines represent the intra-layer connections. In (**a**) we show the classical view of the two spreading processes, separating and constraining each of the spreading processes to only one of the layers. In (**b**) we illustrate our model in which we analyze the coevolution of both processes through the multiplex.

We start from the key assumption that each entity or node in the multiplex network has a different awareness, due to the action of both endogenous (Big Five personality traits, socioeconomic factors data) and exogenous factors (disease data, Google Trends data) and, as a consequence, each node will be heterogeneous susceptible to the disease spreading. The endogenous factors by definition are those linked directly to node’s internal characteristics. On the other hand, the exogenous factors are those corresponding to features that have external sources to the node, and depend on the network and the kind of disease. In particular, in our model, we assume that the transition probability from the susceptible state to the infected state represents the probability of being infected, given the spread of an epidemic disease on multiplex network. We consider two coevolving processes on multiplex network (see Fig. 1). The first is the process of epidemic spreading, indicated by *S*^*h*^*i*^*p*^*IR*, which is a variant of the SIR model of epidemic diseases^1^,^2^, where *S*^*h*^ indicates the heterogeneous susceptible state, *i*^*p*^ is the preventive isolation state, thus a given node is either heterogeneous susceptible to the disease, state *S*^*h*^, or preventive isolated, *i*^*p*^. Then, a node can become infected, *I*, and recover (*R*) from infection. The preventive isolation represents a strategy to delay or avoid the transition into the infected state, then choosing properly the isolation period, it allows reducing the infection rate or avoid the transition to infected if the network has already recovered. The selection of the nodes to be preventively isolated depends on structural parameters, the awareness of the nodes and the socioeconomic factors. The second spreading process is also a SIR-like model, which is an extension of the *UAU* model^22^, indicated by “Unaware - Aware - Faded” (*UAF*), where the state *U* represents the unaware condition of nodes in the network, while *A* is the aware state so that nodes start to raise their attention on epidemic spreading, realizing the risk associated with epidemics. The Faded state (*F*) represents a condition where nodes, once become aware of epidemics, tends to fade their attention with time, until it completely vanishes. If a node reaches this state, unless already recovered from disease, it will result more susceptible to infection, because it does not exploit and update its acquired awareness. Our epidemic model, indicated by *S*^*h*^*i*^*p*^*IR*, “Heterogeneous Susceptible - (Preventive Isolation) - Infected - Recovered”, is expressed diagrammatically, in terms of reaction-diffusion process^1^, as follows:


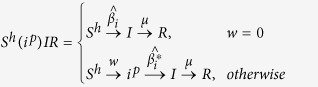


The epidemic model is based on the assumption of heterogeneous susceptibility, *S*^*h*^, that is each node has a different susceptibility to a disease propagating on the network due to the various socioeconomic factors and awareness. This means that we have different values of 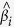, which measures the probability that a node is infected on the multiplex, given that a Zika and other mosquito-borne viruses is propagating with a certain infection rate *β* characterizing the disease, and at least one of its neighbors has already being infected. This hypothesis allows to assert that if there is at least one neighbor infected, it means that the node, most likely, is located in an environment with a high risk of transport of the infection. This concept of infection is consistent with the assumptions deriving from the nature of Zika virus^55^. Moreover, the diagrammatic representation of our model sheds light on its duplex nature linked to include or not the preventive isolation strategy. In fact, if we do not consider preventive isolation, each node, with differently susceptible, will become infected with an infection rate on the multiplex, defined as follows:


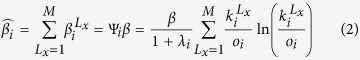


where the heterogeneity factor is defined as follows:


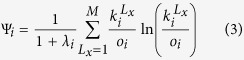


It encloses in its definition the rate of awareness, *λ*_*i*_, and the entropy of the multiplex degree^32^, that represents a parameter to describe the distribution of the degree of node *i* among the various layers. Therefore, *ψ*_*i*_ will depend not only on the different rates characterizing each of spreading processes, but also on the node degree and its distribution on the multiplex. In the definition of entropy of the multiplex degree^32^, *o*_*i*_ and *k*_*i*_ are the overlapping degree and degree centrality of the node *i* in the layer *L*_*x*_. We introduce an extension of the definition of the infection rate, indicated by 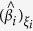, where *ξ*_*i*_ is a socioeconomic factor, calculated for each node as a global measure of resilience of the node in the multiplex according to socioeconomic factors impacting the epidemic and awareness dynamics. In particular, this parameter affects the awareness *λ*_*i*_ of the nodes in the network and, consequently, the infection rate of the nodes, as follows:


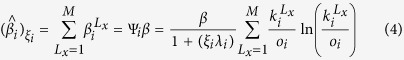


Some indicators and measures have been introduced to evaluate the resilience of a community, and identify the critical dimension of a system. Among socioeconomic factors which influence the resilience, such as social capital, education, language, governance, financial structures, culture, and workforce, in our model we consider some of them, and we aim to understand their role and impact on awareness and epidemic dynamics. The process of awareness spreading, *UAF*, is modeled as follows:


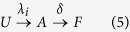


where *λ*_*i*_ represents the rate of awareness of the node *i* in the multiplex as we assume, without loss of generality, that nodes, through the various layer of the multiplex, keep the same features and awareness. We assume that the theoretic distribution of *λ*_*i*_ derives from endogenous and exogenous factors that, in the data-driven modeling (see details in Methods), will be extracted from real data. The parameter *δ* represents the fading rate of attention on current epidemics. It is important to underline how it does not correspond to the loss of awareness^56^, but only to a temporary decay of attention or interest towards epidemics.

### Preventive isolation, Centrality and Awareness

The preventive isolation strategy consists of isolating in advance a set of nodes chosen according to their centrality and awareness values. Nodes are isolated with a probability *w*_*i*_, so that each node has a certain probability of being isolated from the network. Following the diagrammatic representation of the model, if *w*_*i*_ is not null, it temporally excludes a first transition to the status Infected, but does not rule out a future infection in function of the timings of the two spreading processes. We assume that: *t*_*w*_ < *t*_*r*_ *then* 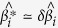, where *t*_*w*_ is the preventive isolation period, *t*_*r*_ is the recovery period, after which every nodes recovers from infection. It means that, given the nature of the virus taken into account in the data-driven modeling, i.e. the Zika virus, the isolation period is less, but not negligible, compared to recovery period. This condition represents a link between the two dynamics of the two spreading processes co-evolving in the multiplex network. To define and identify the set of nodes to be preventively isolated, we introduce a social network approach, considering a scale-free network for each layer of the multiplex network^57^, and taking into account centrality and awareness measures in a multiplex structure^37^. In our model, centrality is calculated using the eigenvector-like centrality measure, which allows to include the concept of influence in our analysis. We consider a multiplex network 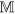, formed by *M* layers and, differently from ref. 23, in both layers the two spreading processes coevolve. All nodes represent the same entities in both layers, but the connectivity patterns are different in each of them. For simplicity, in our work, we consider a multiplex network with *M* = 2 layers and a population of *N* = 1000 nodes. For each layer *L*_*x*_, with 1 ≤ *x* ≤ *M*, we introduce the adjacency matrix, denoted by 
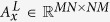
, then we can define the adjacency matrix of the multiplex, denoted by 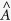:


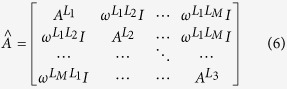


where 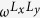 is defined as the strength of the inter-layer interaction between two generic layers *L*_*x*_ and *L*_*y*_ and represent the elements of the inter-layer matrix 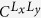. Note that we consider a symmetric interaction measure between two distinct layers (*M* = 2), that is 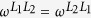, and hence: 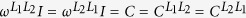. The introduction of the strength of inter-layer interaction demonstrates how in our definition of eigenvector-like centrality we assume that each layer has a different centrality measure. We consider the situation where the influence among layers is heterogeneous. Given a multiplex network 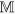 and an influence matrix 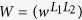, we define the global heterogeneous eigenvector-like centrality of 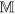
34,37. To identify the set of nodes to whom applying the preventive isolation strategy, we need also an awareness measure in the multiplex structure. To this aim, we define a vector of awareness, whose elements are the rates of awareness of each node, weighted by the socioeconomic factor *ξ*_*i*_, as follows:


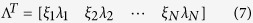


where 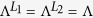, since the awareness is identical for a node in both the layers of the multiplex network, that is the awareness is invariant among layers and for all the interactions among layers. For each layer *L*_*x*_, we define the matrix 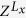, as the Hadamard product between the awareness matrix Λ and the adjacency matrix of the layer, 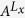, as follows:


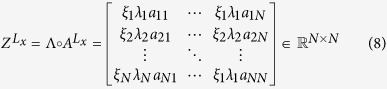


Note that 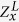 degenerates in the adjacency matrix 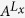 if there is no heterogeneity among nodes. In this case, *λ*_*i*_ would be all equal to 1 (homogeneous awareness) with any difference among nodes. To get an overall measure which includes both the concepts of centrality and awareness in the multiplex structure, we need to evaluate the global heterogeneous eigenvector-like centrality and awareness of the multiplex 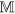, defined as a positive and normalized eigenvector 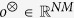 (if it exists) of the matrix:


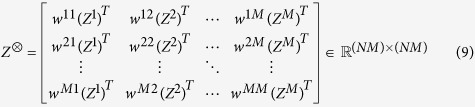


where *Z*^⊗^ is the Khatri–Rao product of the influence matrix *W* and *Z*^*T*^^37^. For each node, we define an overall measure of its centrality and awareness, denoted by *θ*_*i*_, in the multiplex network 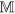. Θ is a column vector of size *N*, which includes all the measures *θ*_*i*_. It allows to quantify the overall weight, in terms of centrality and awareness, of each node in the multiplex 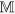, as follows:


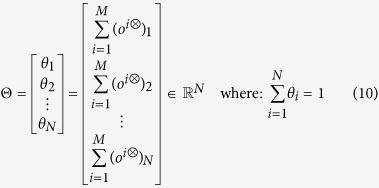


Based on this overall measure of centrality and awareness, we will select a set of nodes to be preventively isolated, associating a different probability of isolation to nodes on each layer of the multiplex.

### Simulation Results

**Figure 2:**
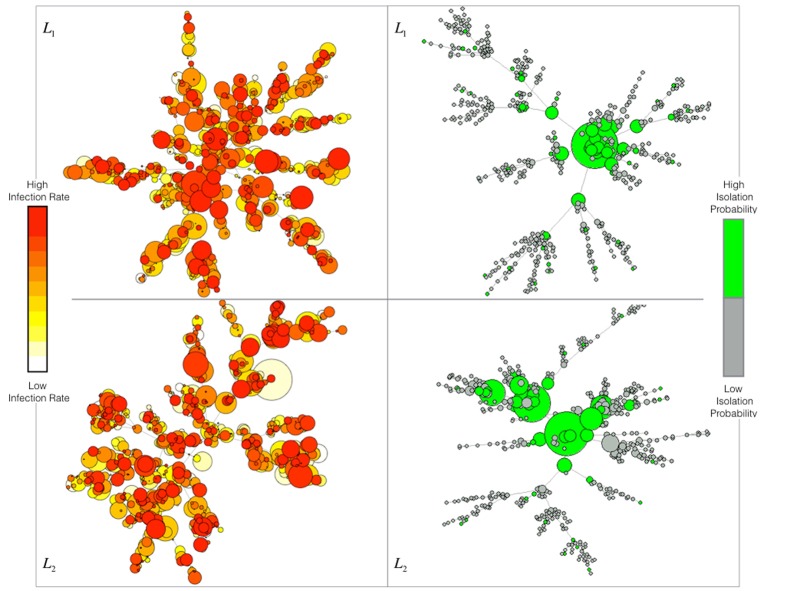
Heterogeneous susceptibility and preventive isolation on multiplex network. We show the heterogeneous distribution of the susceptibility and awareness on the two layers of the multiplex network. In the top-left and in the bottom-left panels, we show the distributions on layer *L*_1_ and *L*_2_ respectively, where the color, ranging from ‘white’ (lowest values) to ‘red’ (highest values), corresponds to the nodes’ infection rate of our model, 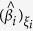. The nodes’ size represents the awareness *λ*_*i*_ of each node *i* in the multiplex. In the top-right and bottom-right panels, we illustrate the preventive isolation strategy and the identification of the set of nodes, green-colored, to be isolated from each layer of the multiplex, according to our model.

**Figure 3:**
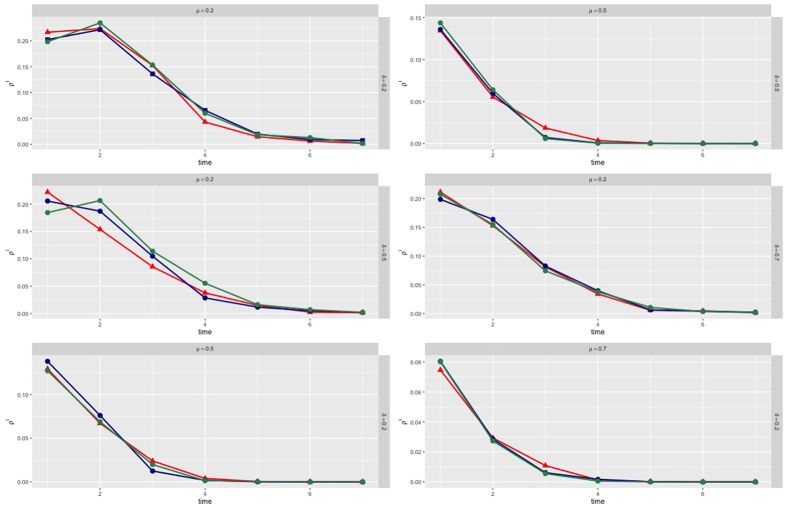
Density of infected nodes over time. We illustrate the temporal evolution of the density of infected nodes *ρ*^*I*^ according to our model, considering the variation of both recovery and fading rates, (*μ, δ*), and the socioeconomic factor *ξ*_*i*_. ‘Red’, ‘blue’ and ‘green’ lines correspond to the lowest, medium and highest levels of *ξ*_*i*_, respectively.

**Figure 4:**
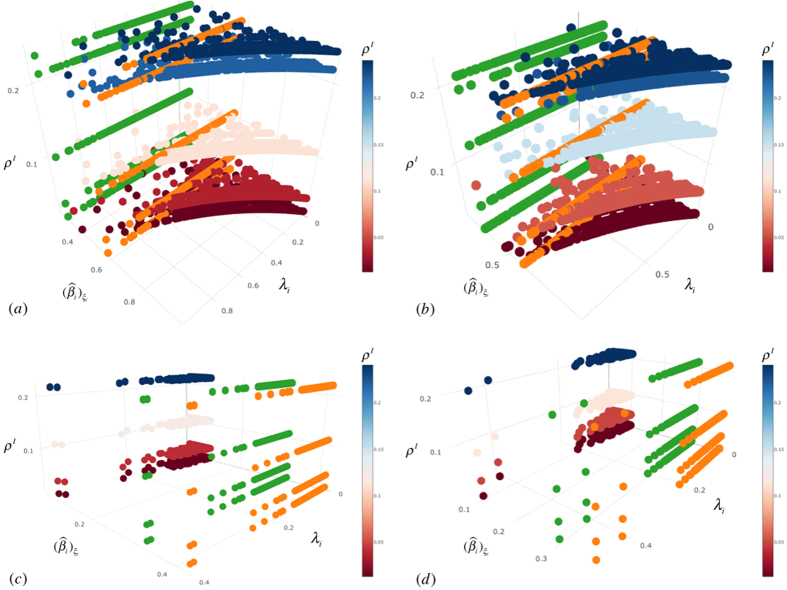
Phase diagrams - *λ*_*i*_, 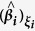, *ρ*^*I*^. Plots in the various panels show the density of infected nodes *ρ*^*I*^ (ranging from ‘red’ to ‘blue’) in function of awareness *λ*_*i*_ and infection rate 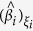, and we shed light on the comparison between the epidemic threshold in the homogeneous susceptibility case (‘green’) and the epidemic threshold derived from our model (‘orange’). In the top panels, (**a**) and (**b**), we illustrate the phase diagrams without preventive isolation strategy in the two cases of high and low values of *ξ*_*i*_, respectively. In the bottom panels, (**d**) and (**e**), we show the corresponding phase diagrams with preventive isolation strategy for the same values of *ξ*_*i*_, respectively. Plots are obtained using MMCA method and MC simulations.

Simulations have been conducted choosing a multiplex network with *M* = 2 layers, where each layer is modeled as a scale-free network^57^ with *N* = 1000 nodes. From Fig. 2, we can observe how the heterogeneous distribution of the infection rate characterizes our model, depending on the awareness and the structural parameters of the multiplex network, considering the features of each node regarding the epidemic spreading. Each node owns a distinct consciousness and then reacts in a different way to the disease, producing a different susceptibility. We also show the set of nodes chosen to be preventively isolated according to their isolation probabilities, where the highest values are assigned to those nodes having a key role and influence on the multiplex structure but, at the same time, a low awareness about infection. Given that isolation is a temporary separation of the node from the network, when a node is isolated from one layer, it will be also isolated in all the multiplex network. In each plot of Fig. 3, curves correspond to the different values of the socioeconomic factors, and show how the number of infected nodes depends on the economic healthy of the nodes. Following the assumptions of our model, the best case is the last one as expected, where we have a high recovery rate and a low fading rate, meaning that nodes recover with a high probability and their attention on disease fades slowly over time. In this case, we do not notice substantial shifts among curves and, as expected from our model, awareness has a stronger impact on the coevolution dynamics than the socioeconomic factors. The worse case corresponds to the top-left plot, where there is a low recovery and fading rate. The underlying reason is that in a real-world scenario, nodes have a high level of attention on the disease propagation, even because only a few nodes have already recovered from disease. The latter case is even worse than the plot obtained with a high fading rate and low recovery rate, since the increasing of faded nodes could also be linked to a major number of nodes in the recovered state which have already lowered their attention on disease (see details in Model). In Fig. 4, the infection rate derives from heterogeneous susceptibility introduced in our model (see equation 4). The awareness is distributed among nodes in the whole multiplex network, meaning that each nodes keeps the same awareness rate in both layers. Both in isolation and without isolation, the heterogeneity allows to delay the epidemic outbreak in comparison with the homogeneous case (where nodes are assumed with the same susceptibility), as highlighted by the epidemic thresholds in both cases. The remarkable difference is due to the dependence of the heterogeneous susceptibility on the awareness of nodes, able to strengthen the resilience of each nodes towards infection. In other words, a node, aware of disease propagating in the network, can take some prevention measures, lowering its susceptibility. Furthermore, the figure highlights the effect of socioeconomic factors on the epidemic threshold: ranging from highest to lowest values of *ξ*_*i*_, both with and without isolation, we observe how the two epidemic thresholds are more and more close to each other. This is due to the joint and striking role of heterogeneity and awareness in delaying the epidemic outbreak, which has a minor impact on nodes with critical economic conditions. The more is the economic unhealthy of the nodes, the more it becomes crucial increasing the nodes’ awareness. In the case of preventive isolation strategy, the epidemic thresholds are shifted and the distribution of densities of infected nodes, in relation with the values of 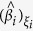 and *λ*_*i*_, results bounded. The phase diagrams obtained by applying the preventive isolation strategy represent idealistic configurations, in fact preventively isolating and quarantining nodes in the considered temporal window *t*_*w*_, the epidemic outbreak will be not reached as expected choosing the ‘best’ nodes to be isolated. Considering a long-time span dynamics, where awareness could decrease in a dramatic way, this isolation strategy does not avoid a possible outbreak. Since it may result wasteful, it should be more convenient to isolate only a fraction of these nodes, while keeping under control the others.

**Figure 5:**
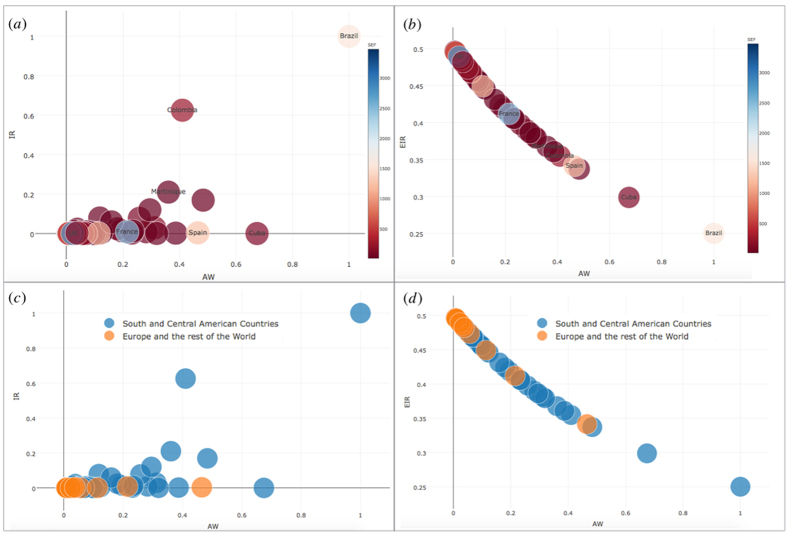
Data-driven analysis in the plane *λ*_*i*_ -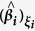. In (**a**) and (**b**), we show the trend of 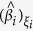 (*IR*) (**a**) and *EIR* (**b**) according to *λ*_*i*_ (*AW*), resulting from data-driven approach and our model, respectively. The color, ranging from ‘red’ to ‘blue’ indicates the socioeconomic factor *ξ*_*i*_ (*SEF*). In (**a**), *IR, AW* and *SEF* are derived directly from data, while in (**b**) we evaluate *EIR* according to our model in function of *AW* and *SEF* data-driven measures. In (**c**) and (**d**), we shed light on the difference between South and Central American countries (‘blue’) and Europe and Rest of the World (‘orange’).

**Figure 6:**
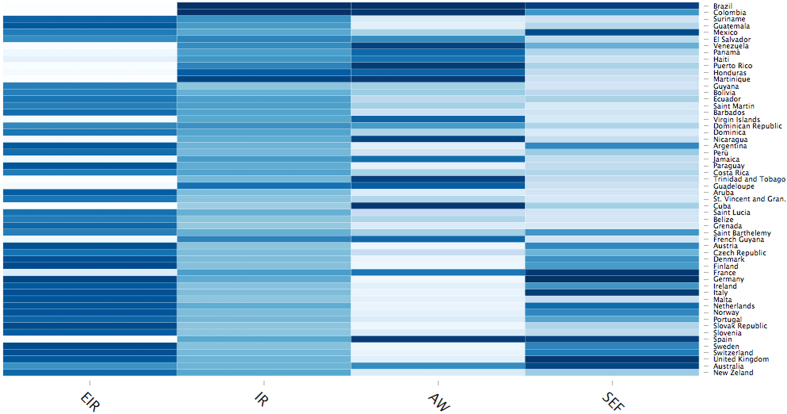
Heatmap of data-driven analysis. In the heatmap, with colors ranging in the blue scale, where *AW* and *SEF* are derived from data, we show the infection rate (*EIR*) evaluated following our model in comparison with the infection rate (*IR*) derived directly from data, observing the different values among the various countries.

In Fig. 5 we exhibit the data-driven approach results referred to countries, chosen according to the data on Zika virus (see details in Methods). Each country constitutes a node *i* in the multiplex network and the overall population *N* is equal to 56. Plot (a) is obtained observing infection rate (*IR*), awareness rate (*AW*) and socioeconomic factors (*SEF*) data. We can observe how nodes mostly have low *IR* and *AW* rates because less or almost not involved in the Zika outbreak risk yet. The situation is totally different in Brazil, which has high values of *IR* and *AW*. In (b), we maintain data-driven *AW* and *SEF* as inputs of our model and, starting from these values, we evaluate the ‘expected infection rate’, indicated by *EIR*, as outcome based on our model (see details in Model). There is an evidence that Brazil has a *EIR* lower than in (a), since we now consider the awareness’ weight of the country on Zika virus in evaluating *EIR*. Hence, similarly, the other countries have a higher *EIR* than in (a), because less aware of Zika virus and then, on average, more susceptible against it. *SEF* acts in conjunction with awareness, but the latter has a stronger impact on *EIR* and thus it encloses a real social value, to be spread properly in countries having economic critical issues. The data-driven trend showed in (b) is in line with the theoretical plot illustrated in Fig. 4. In (c) and (d), it is interesting to see how, although in Europe there are still a few cases of infection in comparison with Americas, it shows a high *EIR* due to its low interest and awareness on Zika virus. Moreover, the figure suggests how awareness should be kept at an high level since extraordinary events, such as the Rio Olympics, may trigger or accelerate unexpected dynamics even in countries which have never been involved in infection. This figure shows the trend, while the cumulative statistics with the single values of the estimators for each state, is reported in Supplementary Information (see Supplementary Table S1). In Fig. 6, we exploit an heatmap to show clearly the sharp contrast between *IR* and *EIR* values, in line with our model, better highlighting the singularities of some countries.

## Methods

### Dynamic Microscopic Markov Chain Approach

**Figure 7:**
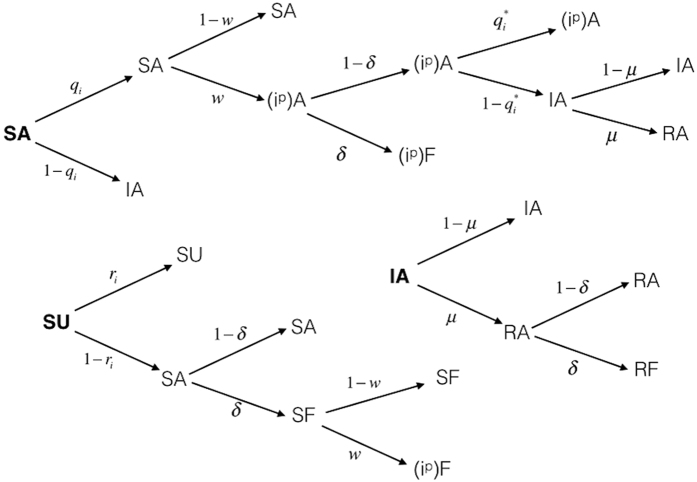
Probability tree for the transitions of states in the model. The states include the possible initial states - *SA, SU* and *IA* - and the arrows indicate the transition probabilities from one state to all the possible subsequent states.

To analyze the coevolving dynamics of both epidemic and awareness spreading on top of the multiplex network, we exploit the Dynamic Microscopic Markov Chain Approach (MMCA). At the initial stage, a state probability is assigned to each node describing its initial state. As a result of the coevolution of the two dynamical processes, it is worth noting that each individual in this multiplex network can only be in one of the three kinds of states: susceptible and unaware (SU), infected and aware (AI), and susceptible and aware (SA). In Fig. 7, the MMCA method is illustrated using a probability tree, which depicts at each time step, the possible states and their transition in our model. It is important to note that there are some states, represented in the diagrammatic models of the two spreading processes (see (1) and (5)), which are not reachable or do not exist. For example, the state *i*^*p*^*U* does not exist since, if a node has been isolated, it cannot be in the Unaware state (U) in fact, after being isolated preventively from the network, it knows that it could be a potential spreader of the disease. Similarly, the state *IU* (Infected Unaware) cannot exist, since an infected node will be surely aware of the epidemics. For the same reasons, the state *RU* (Recovered Unaware) does not exist as it has already recovered from an infection that it knows, then it will be surely aware of it. In other words, following the dynamics of our model, a node, which is has been infected or isolated or recovered, surely knows about epidemics and hence it cannot be unaware of the epidemic spreading in the network. Moreover, we observe that its awareness can derive from any of the two layers in the multiplex network. In the transition tree, roots represent the possible state at the initial state (time step *t*), while leaves of each transition constitute all the possible states at next time step *t* + 1. The transition arrows are labeled with the corresponding probabilities vary in function of the time step and depend on the actual state of the node. For simplicity’s sake, the time dependency is not illustrated in the transition tree. Since at initial time step *t* each node *i* can only be in one of the three states, we denote the probabilities of the three states as 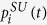, 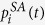, 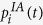 respectively. Furthermore, we define: *q*_*i*_(*t*), probability for node *i* not being infected at time step *t*; 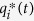, probability for node *i* not being infected at time step *t* after its preventive isolation; and finally *r*_*i*_(*t*), probability for unaware node *i* staying unaware at time step *t*. These probabilities are defined as follows:


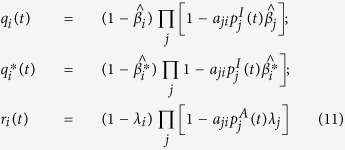


where *a*_*ij*_ are the elements of the adjacency matrix of each layer of the multiplex network. The following MMCA equations represent the probability of each node of being in one of the states at time step *t* + 1:


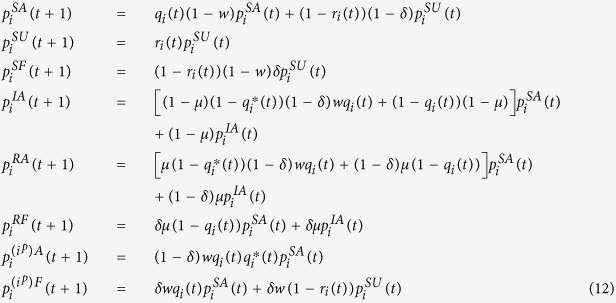


To calculate the epidemic threshold, we need to investigate the steady state solution of the system constituted by the previous equations. When time *t* → +∞, there exists an epidemic threshold *β*_*C*_ for the two coevolving dynamical processes, which means that the epidemic can outbreak only if *β* ≥ *β*_*C*_. In the steady state, the probabilities of states fulfill the conditions:


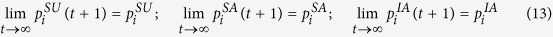


The epidemic threshold is given by the order parameter *ρ*_*i*_ which corresponds to the density of infected nodes in the system, and it is given by:


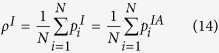


Thus, starting from equation 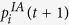 (see equation (12), at steady state:


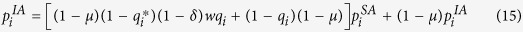


Since around the epidemic threshold *β*_*C*_, the infected probability is close to zero 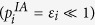, the probabilities of being infected, *q*_*i*_(*t*) and 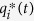, can be approximated as follows:


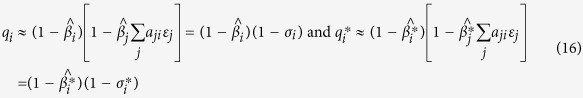


where:


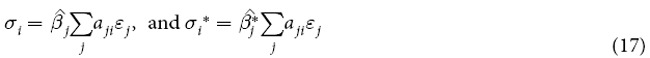


Furthermore, close to the epidemic onset we have: 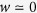, thus the probability of not being infected after isolation is much greater than the same probability without isolation: 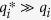. Around the epidemic threshold, starting from the assumptions of our model, the fading rate approximately equal to zero: 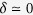. Therefore, inserting these approximations into equation (15) and omitting higher order items, equation (15) is reduced to the following form:


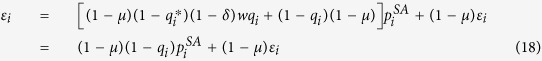


Introducing the expressions of *q*_*i*_ and *σ*_*i*_, we find:


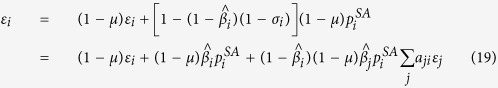


From equation (19), by analyzing the probability 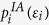, we can get:


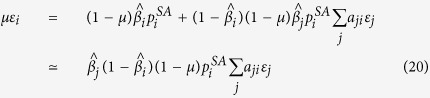


since the first term in the right hand side of the equation (20) is negligible than the second one, given that 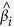, close to the epidemic threshold, is very small. Thus, the previous equation will be:


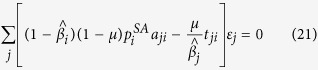


where *t*_*ji*_ are the elements of the identity matrix. By defining the matrix *H*, whose elements are given by:


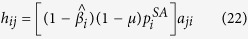


the equation (22) has nontrivial solutions if and only if 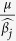 is the eigenvalue of matrix *H*. Consequently, the epidemic threshold 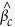 is the one which satisfies 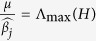, where Λ_max_ (*H*) is the largest eigenvalue of the matrix *H*, and we get:


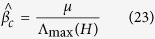


### Data-Driven Analysis

Information about awareness, disease and socioeconomic factors can be obtained in several ways and also exploiting online tools. In our model, we take into account *N* = 56 states across South America, Europe and Oceania, representing the nodes in the multiplex network and all the collected data are referred to this set of nodes. We observe data in a temporal window from April 2015 to June 2016, chosen according to the most relevant temporal dynamics of the Zika virus. Data regarding awareness are collected from heterogeneous data sets, each representing one of the aspects included in the model. Therefore, we consider data from the Big Five personality traits^58^, where the personality traits are reported across major regions of the world, and we choose the entries corresponding to our set of nodes. To evaluate the time evolution of awareness and set up a measure related to the interest on Zika virus, we look at Google Trends, keeping track the total search-volume of the term ‘Zika’ and semantic associated terms for each of the regions of the world included in our model. To extend our measure of awareness taken from data, we consider the online informal sources and real-time emerging public health threats of Zika virus outbreak^59^. Awareness is an aggregated measure of these sources. The measure of infection rate is obtained from the data set “World Zika virus Outbreak 2016” of the data repository Knoema^60^, considering both suspected and infected cases. The socioeconomic factors data are referred to the GDP (Gross Domestic Product) values from data set “Global GDP 2016”^60^, as a measure of the well-being and economic healthy of the considered countries.

## Discussion

Our study has proposed a novel model to explore and quantify the coevolution of epidemic and awareness spreading on a multiplex network, with the introduction of a preventive isolation strategy and adding a data-driven evaluation from multiple sources and types of data of a real emerging Zika virus on a population of nodes, representing countries involved in the epidemic outbreak. Our idea has been to include realistic multiple social critical aspects in the epidemic spreading on multiplex network. As observed in ref. 61, there is a direct relationship between the economic crisis and the epidemic growth rate since more and more people fail to get treatment, to buy drugs, or take the right prevention measures. All these issues, together with the nodes features, both psychological and social, influence the dynamics of the two spreading processes on the multiplex network. One of the main targets has been to analyze and weigh all these aspects, quantifying and reasoning about the strong and nontrivial impact on the resulting epidemic dynamics. Findings have highlighted the striking role of heterogeneity and awareness in the epidemic spreading dynamics under different socioeconomic conditions as expected. Awareness, even if it is not able to totally control the epidemic spreading, acts on susceptibility, increasing the resilience of nodes and delaying the epidemic outbreak. This has been even more crucial under critical economic conditions. The delay in the epidemic outbreak encourages a better understanding of epidemic from health management organization, giving them the opportunity to apply some countermeasures to control and evaluate the comorbidity risk with other diseases^43^. In future, we aim at performing a preventive isolation strategy as a result of comorbidity factors, detected at a certain time step in function of the epidemic outbreak. Once ascertained, this comorbidity risk^62^ should be a control parameter, tuning and timing the isolation strategy according to a comorbidity risk temporal window. Moreover, considering the dynamics of infection characterizing Zika virus, due also to sexual contacts, we also target to explore the possible analogies between Zika and HIV infection. Furthermore, we aim to deepen the intangible impact of the economic complexity in evaluating the competitiveness of various countries^63^ and their impact on the epidemic spread.

## Additional Information

**How to cite this article**: Scatà, M. *et al*. The Impact of Heterogeneity and Awareness in Modeling Epidemic Spreading on Multiplex Networks. *Sci. Rep.* **6**, 37105; doi: 10.1038/srep37105 (2016).

**Publisher's note**: Springer Nature remains neutral with regard to jurisdictional claims in published maps and institutional affiliations.

## Supplementary Material

Supplementary Information
