# Supplementary material for: The Impact of Heterogeneity and Awareness in Modeling Epidemic Spreading on Multiplex Networks
Source: Sci Rep. 2016 Nov 16;6:37105. doi: 10.1038/srep37105 (PMC5111071; doi:10.1038/srep37105)
Supplement: Supplementary Information [file srep37105-s1.pdf]

Supplementary File

The Impact of Heterogeneity and Awareness in Modeling Epidemic Spreading on Multiplex Networks

Marialisa Scatà[1,*], Alessandro Di Stefano[1], Pietro Liò[2], and Aurelio La Corte[1]

[1]University of Catania, Dipartimento di Ingegneria Elettrica, Elettronica e Informatica, Catania, 95125, Italy

[2]University of Cambridge, Computer Laboratory, Cambridge (UK), CB3OFD, UK

* corresponding author (lisa.scata@dieei.unict.it)

[+]these authors contributed equally to this work

Supplementary Table S1. Cumulative statistics of the model's estimators. Columns contain the single values of the estimators for each state, where: IR - infection rate, EIR - expected infection rate, AW - awareness rate, SEF - socioeconomic factors. In bold we indicate some of the most relevant cases in terms of EIR values with regards to IR and the other estimators.

State	EIR	IR	AW	SEF
**Brazil**	**0.2500340**	**1.000000e+00**	**0.999727826**	**1534.800**
**Colombia**	**0.2710183**	**6.253270e-01**	**0.844893882**	**253.200**
**Suriname**	0.4704395	2.175320e-02	0.062835940	4.300
**Guatemala**	0.4813405	1.186418e-02	0.038765744	68.100
**Mexico**	0.4443512	3.712512e-03	0.125235962	1082.400
**El Salvador**	0.4049172	7.880169e-02	0.234820211	27.300
**Venezuela**	0.3536360	2.917162e-02	0.413883161	185.600
**Panamà**	0.3363451	8.957590e-03	0.486568392	55.800
**Haiti**	0.4020118	1.407055e-02	0.243744538	8.200
**Puerto Rico**	0.3156617	7.709737e-02	0.583974020	99.700
**Honduras**	0.2733113	1.692562e-01	0.829415861	20.600
**Martinique**	**0.2500000**	**2.098824e-01**	**1.000000000**	**9.610**
**Guyana**	0.4126026	3.963535e-05	0.211819665	3.300
**Bolivia**	0.4152591	8.323424e-04	0.204067570	34.000
**Ecuador**	0.4590018	9.380367e-03	0.089320272	94.000
**Saint Martin**	0.4447417	8.554631e-03	0.124248098	1.394
**Barbados**	0.4647575	5.205443e-03	0.075829793	4.500
**Virgin Islands**	0.3997423	2.345092e-03	0.250805925	1.095
**Dominican Republic**	0.3997373	2.431629e-02	0.250821402	71.400
**Dominica**	0.4111928	4.452372e-03	0.215974474	500
**Nicaragua**	0.3082378	1.942132e-03	0.622124426	12.900
**Argentina**	0.4833764	5.945303e-04	0.034390521	437.900
**Perù**	0.4691783	5.152596e-04	0.065692933	178.600
**Jamaica**	0.3694701	1.247192e-02	0.353289386	14.100
**Paraguay**	0.4879156	1.869468e-03	0.024767436	26.800
**Costa Rica**	0.4452321	5.390408e-03	0.123009860	56.900

**Trinidad and Tobago**	0.3829086	5.482891e-04	0.305794601	23.800
**Guadeloupe**	0.3562823	1.202206e-01	0.403381673	9.740
**Aruba**	0.4756167	1.123002e-04	0.051266642	2.516
**St. Vincent and Gran.**	0.4354099	5.284714e-05	0.148343253	800
**Cuba**	**0.3273070**	**6.605892e-06**	**0.527617842**	**121.000**
**Saint Lucia**	0.4577884	8.984014e-04	0.092207589	1.400
**Belize**	0.4477796	3.302946e-05	0.116620791	1.800
**Grenada**	0.4636050	1.321178e-05	0.078504268	1.000
**Saint Barthelemy**	0.4179632	1.037125e-03	0.196277540	255.000
**French Guyana**	0.3710919	5.897741e-02	0.347375220	3.810
**Austria**	0.4897120	3.302946e-05	0.021008174	384.400
**Czech Republic**	0.4729923	1.189061e-04	0.057099709	185.300
**Denmark**	0.4917725	1.123002e-04	0.016730378	301.800
**Finland**	0.4937861	6.605892e-05	0.012584131	234.600
**France**	**0.3674349**	**7.966706e-03**	**0.360785281**	**2464.800**
**Germany**	0.4943376	1.255120e-03	0.011454536	3467.800
**Ireland**	0.4805012	9.908839e-05	0.040580040	254.600
**Italy**	0.4940456	2.972652e-04	0.012052264	1848.700
**Malta**	0.4894987	3.302946e-05	0.021453265	10.300
**Netherlands**	0.4894285	1.189061e-03	0.021599692	762.500
**Norway**	0.4942394	3.170828e-04	0.011655522	366.900
**Portugal**	0.4743023	4.161712e-04	0.054179909	205.100
**Slovak Republic**	0.4946297	3.302946e-05	0.010857145	89.800
**Slovenia**	0.4859688	3.302946e-05	0.028872662	43.800
**Spain**	**0.2568729**	**3.514335e-03**	**0.946487768**	**1242.400**
**Sweden**	0.4961228	6.605892e-05	0.007815047	512.700
**Switzerland**	0.4927311	4.359889e-04	0.014752201	651.800
**United Kingdom**	**0.4856202**	**7.596776e-04**	**0.029611242**	**2761.000**
**Australia**	0.4278671	6.209539e-04	0.168587026	1200.800
**New Zeland**	0.4725521	2.721628e-03	0.058084496	169.900
